# Eight years’ experience with a Medical Education Journal Club in Mexico: a quasi-experimental one-group study

**DOI:** 10.1186/s12909-015-0499-7

**Published:** 2015-12-14

**Authors:** Melchor Sánchez-Mendiola, Daniel Morales-Castillo, Uri Torruco-García, Margarita Varela-Ruiz

**Affiliations:** Department of Medical Education Research, Secretariat of Medical Education, UNAM Faculty of Medicine, Ave. Universidad 3000, México City, 04510 D.F. México México

**Keywords:** Journal club, Continuing professional development, Evidence based medical education, Postgraduate medical education, Faculty development

## Abstract

**Background:**

A time-honored strategy for keeping up to date in medicine and improving critical appraisal skills is the Journal Club (JC). There are several reports of its use in medicine and allied health sciences but almost no reports of JC focused on medical education. The purpose of the study is to describe and evaluate an eight years’ experience with a medical education Journal Club (MEJC).

**Methods:**

We started a monthly medical education JC in 2006 at UNAM Faculty of Medicine in Mexico City. Its goal is to provide faculty with continuing professional development in medical education. A discussion guide and a published paper were sent 2 weeks before sessions. We reviewed the themes and publication types of the papers used in the sessions, and in June-July 2014 administered a retrospective post-then-pre evaluation questionnaire to current participants that had been regular attendees to the JC for more than 2 years. The retrospective post-then-pre comparisons were analyzed with Wilcoxon signed-rank test. Effect sizes were calculated for the pre-post comparisons with Cohen’s *r*.

**Results:**

There have been 94 MEJC sessions until July 2014. Average attendance is 20 persons, a mix of clinicians, educators, psychologists and a sociologist. The articles were published in 32 different journals, and covered several medical education themes (curriculum, faculty development, educational research methodology, learning methods, assessment, residency education). 22 Attendees answered the evaluation instrument. The MEJC had a positive evaluation from good to excellent, and there was an improvement in self-reported competencies in medical education literature critical appraisal and behaviors related to the use of evidence in educational practice, with a median effect size higher than 0.5. The evaluation instrument had a Cronbach’s alpha of 0.96.

**Conclusions:**

A periodic Medical Education Journal Club can improve critical appraisal of the literature, and be maintained long-term using evidence-based strategies. This activity is a useful adjunct to the scholarship of teaching.

## Background

Journal Clubs (JC) are a time-honored method of reviewing and discussing the scientific literature in medicine [1, 2]. There is no universally accepted definition of JC, even though this educational modality has been around for more than a century and has evolved substantially during this time. Several published papers about JC in the health sciences define it as “a group of individuals who meet regularly to discuss critically the clinical applicability of articles in the current medical journals” [1]. Some authors have described the origin and history of JC in medicine, apparently Sir William Osler was the first to establish a formal JC at McGill University in Montreal, Canada, in 1875 [1, 2].

Initially the main goal of the JC was to help participants keep abreast of the growing body of medical literature. In the era of paper-only medical journals it was time-consuming and expensive to access the original papers needed to maintain competence in medicine. Over time the focus of JC has moved to teaching critical appraisal skills and evidence-based medicine, while preserving the original objective of helping students, residents and practicing physicians keep up-to-date in the research literature of their field [2, 3]. The appropriate methodology to implement an effective JC in medicine, as well as the factors associated with a successful one, have been extensively reviewed and discussed [3–6].

Even though JC are popular and used frequently in academic health centers and medical schools, there is little published information about JC dedicated to the subject of medical education. There are only three brief case reports with scant data [7–9], and a review paper in the “12 tips” format [10]. This limited amount of information is surprising, since the field of medical education has grown enormously in the last decades, as shown by the large global number of master in health professions education programs, the variety of related research papers and scientific journals, and the rising number of medical education organizations and academic venues [11–13]. There is a need to formally explore the use of JC in medical education settings, to promote the use of educational evidence by clinicians and basic science teachers. Our research question was: does a medical education Journal Club (MEJC) increase self-reported educational research knowledge and skills in our University medical educators? We describe the design, long-term implementation and evaluation of a medical education Journal Club (MEJC) at UNAM Faculty of Medicine in Mexico City.

## Methods

### Setting

The National Autonomous University of Mexico (UNAM) Faculty of Medicine in Mexico City is the largest medical school in the country and one of the largest in Latin America, with more than 7,000 undergraduate students and about 9,000 medical residents. It is a public institution and the largest generator of basic and clinical medical research in Mexico, through its affiliations with major national academic medical centers. Its Postgraduate Studies Division (PSD) is the largest graduate program in Mexico. The PSD offers 78 medical specialty courses, and currently has 8,739 registered medical residents and almost fifteen hundred clinical teachers [14]. The Division has academic staff responsible for faculty development activities, medical education service and research.

### Origin and development of UNAM MEJC

In January 2006, we designed a Journal Club focused on medical education, specifically for the Postgraduate Studies Division. The goals of the MEJC were: to provide continuous professional development to the Division scholars; to discuss the use of research in addressing medical residency educational challenges; to keep up to date in the medical education field; to identify sources of medical education publications and to improve knowledge of medical education research methodology. The MEJC was targeted to PSD academic staff and clinicians responsible for residents’ education from the neighboring academic health centers affiliated with the university.

### MEJC Methodology

The MEJC is a monthly one-hour activity, in a regular time and day. It is organized by the PSD academic staff, and has been led for the last eight years by a pediatrician with a Masters’ degree in Health Professions Education. The PSD office sends a formal invitation and the documents to be discussed to the regular attendees.

The MEJC coordinator selects papers from the medical education literature, relevant to graduate medical education. The selection is informed by requests from regular session attendees and the current educational challenges of the Division. The selected paper and a list of questions designed as discussion guide to promote reflection, analysis and application of the concepts described in the study, are sent by e-mail to the group of potential attendees, 1 to 2 weeks before the session. A core regular population of participants has developed, as well as a smaller, less-regular attendee group. The design, planning and implementation of the MEJC has used several principles and strategies reported in the literature, to increase its chance of success and sustainability [3–6, 15].

### MECJ evaluation

The authors reviewed and classified the papers discussed in the sessions, using a schema that identified papers by research design (quantitative, qualitative, mixed methods), type of article (case report, essay, observational or experimental study, systematic or narrative review), content area (curriculum, teaching, learning, faculty development, assessment, program evaluation, education research), purpose of research [16], type of journal (medical education, clinical or specialty journal, general education journal) and journal title.

We designed a questionnaire to evaluate this academic activity, that included attendees’ demographic data, sessions’ characteristics, and a “retrospective pre-post” section that assessed their self-reported change in medical education literature critical appraisal knowledge and competencies, as well as behavior modification related to their use of the sessions’ concepts in their educational practice. The respondents were asked to provide their best overall estimate for the sessions of each specific aspect explored.

We used a quasi-experimental “retrospective pre-post” methodology (also called “retrospective post-then-pre” design) to control the response-shift bias that frequently occurs in traditional pre-post evaluations, minimizing pre-test over or underestimation [17–19].

“Retrospective pre-post” is a term that is not widely used in clinical research. This method refers to the assessment of learners’ self-reported changes in knowledge, skills, confidence, attitudes or behaviors where both before and after information is collected at the same time. After the educational experience learners are asked to rate their current knowledge, skill, attitude, behavior as a result of the program, and then, to reflect back and rate that same knowledge, skill, attitude, behavior before participating in the program [17–19]. This methodology was described in the 1970’s as a way to minimize the “response shift bias” that occurs in traditional pre-post design. This bias happens when a participant uses a different frame of understanding about an item between the pre and post periods, since learners may not accurately assess their pre-program knowledge or behaviors. At the end of the program, their new understanding of content may affect their response on the post self-assessment, so they are actually responding based on two different frames of reference [20–23].

Research has shown that response shift can mask program effectiveness. The retrospective pre-post design reduces or eliminates response shift bias; compared with the traditional pre- and post design, results from the retrospective post-then-pre design are more consistent with interview data collected from program participants. The retrospective post-then-pre method has been used in many settings and appears to reduce response shift bias across contexts. Responding to both measures at the same time is less burdensome and intrusive for learners, and provides before and after data for each learner [20–23].

The questionnaire also included open items related to suggestions about the MEJC. The instrument was administered to the regular attendees of the MEJC in a voluntary and anonymous fashion. We distributed the questionnaires at the end of the June and July 2014 sessions, but only to the participants that had attended the Journal Club for more than 2 years. The response rate of the current 2014 regular attendees (those that attend more than 80 % of the sessions per year) was 100 %, two participants that are authors of this study did not respond the questionnaire. During the eight-year period, there are some participants that have moved to other institutions, we included only the current participants.

### Data analysis

Data were analyzed with PRISM Version 6 for the Macintosh (http://www.graphpad.com/scientific-software/prism/), obtaining descriptive and inferential statistics. The retrospective pre-post comparisons were analyzed with the non-parametric Wilcoxon signed-rank test. *P* values less than 0.05 were considered statistically significant. Effects sizes for non-parametric Wilcoxon-signed rank test results were calculated for the pre-post comparisons with Cohen’s *r* [24].

### Ethical aspects

The study was in compliance with the Declaration of Helsinki of ethical principles for research involving human subjects. It was approved by the Research Department of the Postgraduate Studies Division, UNAM Faculty of Medicine. Verbal informed consent was obtained. The data were managed anonymously, and analyzed in the aggregate.

## Results

94 MEJC sessions have taken place between 2006 and 2014, about 11 per year. The average attendance is 20, a mix of clinicians, educators, psychologists and one sociologist. Attendance is voluntary, and a certificate of attendance with curricular value in our university is given to the participants every year. The type of articles reviewed cover the breadth of medical education research, mostly related to postgraduate medical education (curriculum development, hidden curriculum, faculty development, educational research methodology –quantitative, qualitative and mixed methods-, learning methods, assessment, systematic reviews, e-learning, among others). The articles’ scope and relevant methodological data are presented in Tables 1, 2, 3, 4.

**Table 1:** Content area of papers discussed in the medical education journal club of the postgraduate studies division, UNAM faculty of medicine

Content area	Teaching and learning strategies	Faculty development, curriculum	Research and EBM education	Resident as teacher	E-learning, informatics	Duty hours, burnout, harassment	Assessment	Education research	Other
*n* = 94	14	11	6	2	8	12	8	14	19

*EBM* = Evidence-based medicine

**Table 2:** Classes of papers discussed in the medical education journal club of the postgraduate studies division, UNAM faculty of medicine

Class of article	Case report	Essay, opinion paper	Observational study	Experimental study	Systematic review	Narrative review	Other
*n* = 94	2	12	23	10	11	30	6

**Table 3:** Research design and purpose of papers discussed in the medical education journal club of the postgraduate studies division, UNAM faculty of medicine. (These data refer to the 52 papers that were actually research studies)

Research design	Quantitative	Qualitative	Mixed methods
*n* = 52	39	11	2
Purpose of research	Descriptive	Justificatory	Clarification
*n* = 52	31	16	5

**Table 4:** Type of journal where the papers discussed in the medical education journal club were published

Type of journal	Medical education	Medical journal	Education journal	Other
*n* = 94	59	32	2	1

The papers discussed in the MEJC were published in 32 different journals, the more frequent sources were: Medical Education = 14 (15 %), Academic Medicine = 13 (14 %), Medical Teacher = 12 (13 %), and Advances in Health Sciences Education = 7 (7 %). The evaluation questionnaire was responded by 22 participants, excluding the MEJC coordinator. The data were captured by a person not involved in the implementation of the MEJC, and analyzed in aggregate form. The demographic data of the participants are outlined in Table 5.

**Table 5:** Demographic data of journal club participants. Demographic data of the medical education journal club participants that responded the evaluation questionnaire, postgraduate studies division, UNAM faculty of medicine (*n* = 22)

Gender	Male = 18 (82 %) Female = 4 (18 %)	
Age (mean ± SD)	60.4 ± 10.5 years (Range 36 to 83)	
Highest degree earned	College = 2 (9 %), Master = 5 (23 %), Medical specialty = 10 (45 %), PhD = 5 (23 %)	
Professional field	Medicine = 19 (88 %), Sociology = 1 (4 %), Psychology = 1 (4 %), Pedagogy = 1 (4 %)	
Medical specialty	Family medicine = 3	General surgery = 2
	Internal medicine = 2	Neurosurgery = 1
	Pathology = 1	Otolaryngology = 2
	Oncology = 1	Epidemiology = 1
	Critical care = 1	Pneumology = 1
	Psychiatry = 2	Rehabilitation
	Pediatrics = 1	medicine = 1
Direct contact with residents in teaching activities	Yes = 17 (77 %) No = 5 (23 %)	
Number of MECJ sessions attended last year (median ± IQR)	9 ± 3	
Formal training in medical education	Diplomate = 10, Masters = 1, PhD = 2, Other = 2, No = 6	

Eight aspects of the MEJC sessions were assessed with a 1 to 9 scale, where the respondents were asked to provide their best overall estimate for each specific aspect of the sessions. The results are shown in Table 6. The majority of these aspects had a positive evaluation.

**Table 6:** Evaluation of Journal Club sessions. Evaluation of the medical education journal club sessions, postgraduate studies division, UNAM faculty of medicine (*n* = 22)

MEJC aspects	Score (median ± IQR)
Facilities/Physical area	9 ± 1
Inadequate = 1–3, Adequate = 4–6, Excellent = 7–9	
Session participants	8 ± 1
Difficult group, negative experience = 1–3	
Not a positive or negative factor for the session = 4–6	
Positive group, improved the experience = 7–9	
Duration of the sessions	6 ± 1
Short = 1–3, Appropriate = 4–6, Long = 7–9	
Amount of learning in the sessions	8 ± 2
Small = 1–3, Moderate = 4–6, Large = 7–9	
Quality of the discussed papers	8 ± 2
Low = 1–3, Medium = 4–6, High = 7–9	
Usefulness of reviewed papers for my teaching practice	8 ± 2
Low = 1–3, Regular = 4–6, High = 7–9	
Usefulness of the printed discussion guide	8 ± 2
Low = 1–3, Regular = 4–6, High = 7–9	
Coordination and guidance of the discussions	8 ± 1
Inadequate = 1–3, Regular = 4–6, Excellent = 7–9	

The participants had to choose a number between 1 and 9 for each feature

*IQR* = interquartile range

The “retrospective pre-post” portion of the questionnaire showed statistically significant increases in knowledge and skills related to critical appraisal of the medical education literature, as well as positive changes in behaviors related to the use of published evidence in their educational practice (Tables 7 and 8). The overall median increase in knowledge score of medical education research concepts and critical appraisal skills was 3 (in a scale of 0 to 10), with a median effect size of 0.80. The instrument’s reliability was 0.96, measured with Cronbach’s alpha.

**Table 7:** Knowledge change in medical education critical appraisal skills. Results of the retrospective pre-post (post-then-pre) knowledge level about topics related to medical education literature critical appraisal skills (*n* = 21)

Topics	Pre median (IQR)	Post median (IQR)	Effect size (Cohen’s *r*)
Critical appraisal of medical education articles	5 (4)	8 (2)*	0.84
Databases and sources of information relevant to medical education	5 (4)	9 (2)	0.86
Systematic reviews in medical education	5 (5)	8 (3)	0.80
Evidence-based medical education	6 (4)	8 (3)	0.78
Research design in medical education	4 (4)	8 (3)	0.82
Qualitative research in medical education	5 (4)	8 (4)	0.78
Statistical methods in education research	6 (6)	8 (4)	0.79
Assessment methods in medical education	6 (4)	8 (2)	0.84
Validity and reliability in assessment	5 (5)	8 (2)	0.76

Scale from 0 = minimum to 10 = maximum

*All pre-post comparisons using Wilcoxon signed-rank test were statistically significant with *p* < 0.001

*IQR* = interquartile range

**Table 8:** Behavior change in the use of evidence in educational practice. Results of the retrospective pre-post (post-then-pre) self-reported frequency of behaviors related to the use of evidence in teaching, before and after the medical education journal club sessions (*n* = 22)

Behavior	Pre median (IQR)	Post median (IQR)	Effect size (Cohen’s *r*)
Reflect on the scientific basis of my teaching activities	3 (2)	5 (1)	0.81
Consult published medical education literature to face educational problems	3 (1)	4 (1)	0.85
Identify the relevant methodological aspects in medical education research papers	3 (1)	4 (1)	0.83
Discuss with the students the scientific basis of our educational activities	3 (1)	4 (1)	0.86
Update my knowledge of medical education	3 (2)	5 (1)	0.85

Scale: 1 = Very rarely, 2 = Rarely, 3 = Occasionally, 4 = Often, 5 = Very often

*All pre-post comparisons using Wilcoxon signed-rank test were statistically significant with *p* < 0.001

*IQR* = interquartile range

## Discussion

This study is the first report that provides a detailed description of the implementation of a journal club dedicated to medical education. It suggests that this scholarly activity may improve knowledge and behaviors related to the teaching activities of medical educators, and promote evidence-based medical education in a medical school context. Traditional JC are ubiquitous in medical schools and health care institutions, this educational modality has a long and rich history in medicine and allied health sciences all over the world [1–3, 5, 15, 25–29]. The flexibility and intellectual challenge provided by JC have stimulated its use not only in general medicine and several medical specialties, but also in nursing, dentistry and other allied health professions [15, 27, 30–35]. JC have diverse goals, like promoting self-directed learning, keeping up-to-date in the published literature, linking published science with practical clinical problems, and recently they are frequently used to teach critical appraisal skills and evidence-based medicine [36–40].

It is interesting and paradoxical that with such extensive dissemination of the JC methodology, there are almost no published papers related to its use in the field of medical education. There are many reports of its use as an educational tool and strategy, but almost none about its utilization for discussing medical education published papers. We did a wide-ranging search in the most important biomedical and educational databases for published paper descriptions and research studies related to medical education journal clubs, and found many papers (171 references) describing JC in medicine and other health professions, but only four related to medical education JC [7–10]. Three are short reports: the first describes the use of JC as a continuing medical education activity for teachers at Universidad Austral in Buenos Aires, Argentina [7]; the second is a description of an advanced education faculty development program for primary care physicians at the Medical College of Wisconsin, USA [9]; and the third is a case report of a faculty development initiative for a major curricular change using the JC as a culture changing strategy, at the University of Virginia School of Medicine, USA [8]. These papers are brief descriptions of their MEJC, and do not include data to evaluate their educational effectiveness. The fourth paper is a set of recommendations published in the Medical Teacher “12 tips” format, where after surveys and interviews with the McGill University Centre for Medical Education scholars, they propose guidelines for a successful medical education journal club [10].

We searched Google with the term “medical education journal club”, and we found many web sites that announce MEJC activities in many medical schools in several countries. We can only speculate on the reasons for this asymmetry in the number of scholarly publications about the JC method in medicine and its limited number in medical education JC, but we think it is important to approach MEJC with a scholarly lens, and report positive experiences that could be relevant to clinicians and medical educators in a variety of settings.

Our study describes a specific medical education journal club methods and strategies, and presents data on self-reported educational effectiveness. There are guidelines for organizing and maintaining a JC, most from clinical JC papers, and one of the consistent recommendations is that these educational events need specific goals and a designated leader/coordinator (which can be one person or a rotating position) with disciplinary expertise, in this case a medical education background. There are no follow-up reports of the published MEJC experiences, so we don’t know if they are currently active, but those papers emphasize that faculty with educational background are important to start and maintain the project, preferably from an office or department of medical education, and that clinicians with educational responsibilities should be involved [7–9]. In our setting the group coordinating the MEJC has been the same for more than 8 years, led by a pediatrician with clinical experience and formal training in health professions education. McLeod and colleagues suggest that the leadership of a MEJC should vary, so every regular participant can have the opportunity of leading the discussion [10]. In any case, the decision of who coordinates the MEJC should be an academic one obtained by group consensus.

Our MEJC has several similarities with the published case reports, like its monthly periodicity (Simpson reported a bimonthly, Centeno monthly and Pollart a biweekly JC), short duration of the sessions, broad variety of articles and content, and high levels of satisfaction in the participants. There are some differences in our approach: we focus on papers that are relevant for residency education; and we choose only one paper for discussion in each session, unlike Simpson’s approach where they review 12 to 15 journals in an hour and choose about eight papers for critical appraisal and follow-up. One of our main goals is to learn medical education critical appraisal skills and research methodologies, and a one-hour session is just enough to discuss one paper appropriately. The variety of topics and types of papers discussed in our MEJC provides a reasonable overview of medical education in residency training. We have emphasized themes like assessment, research education, duty hours and fatigue, faculty development, among others.

We have followed several of McLeod’s recommendations for a successful MEJC: broad representation of participants; visible leadership support (the Director and high-level staff from the Division usually attend); provision of coffee and pastries; consistency and punctuality, every year we have had about 11 sessions, rain or shine; a pleasant and modern physical facility; participants’ enthusiasm and loyalty (more than 70 % of the current attendees have participated since 2006); an stress-free open environment for discussion; focus on methodology and practical implications of educational research in our setting; use of a semi-structured discussion guide with open-ended questions; it is not mandatory but we register attendance, and at the end of the year we provide a valid certificate that is useful in our university recognition system.

Our findings show a high level of satisfaction with the MEJC activity, from the physical setting to more complex aspects like the quality of group discussions (Table 6). Our data show a substantial and statistically significant increase in self-reported knowledge of several domains related to medical education research and evidence-based education (Tables 7 and 8). Cohen’s guidelines for the interpretation of effect sizes expressed as “*r*” are that a large effect is 0.5, a medium effect 0.3, and a small effect 0.1 [24]. The effect size in all our items for knowledge and behavior change is large (>0.5), which suggests an effect that can be educationally relevant [41]. This is apparently the first study that provides educational impact data with a MEJC, and paves the way for other studies. Evidence-based recommendations for JC effectiveness, McLeod and colleagues’ 12 tips for a successful MEJC, and our findings are a reasonable starting point for planning, implementing and developing a MEJC in a medical education setting.

There are some limitations to the study: it is the experience of a single medical school in a developing country, with its inherent external validity and ecological generalizability implications; the data are self-reports, which rises the question of the shortcomings of self-assessment in medicine and the frequent overestimation of one’s abilities. We are aware that there is a need for objective external instruments that measure knowledge and behavior modification, but that was not feasible in our study settings. Nonetheless self-efficacy may be associated with future performance and behavior in some contexts [42, 43], and when individuals transit from the pre-contemplation phase to the contemplation and preparation stages of Prochaska’s transtheoretical model of behavior change, there is a possibility they move to the action phase [44]. Our results suggest that MEJC participants are aware of the need and potential for behavior change and maybe these self-reported increases in knowledge and behavior could be educationally relevant.

We used a quasi-experimental one-group retrospective pre-post research design methodology (also called “post-then-pre”) instead of the traditional one-group pretest-posttest design, because the retrospective strategy is less intrusive, takes less time, and minimizes pretest sensitivity and the response shift bias that can occur as a consequence of over or underestimation of the individuals’ self-perceived changes in knowledge and abilities [17, 18]. On the other hand, in our case several years had passed since we started the JC, so an actual pretest was not feasible.

There are also some limitations to the JC methodology itself, which can explain its limited capacity to produce behavior change in residents and physicians, as has been shown in several studies [5, 27, 38, 45]. It is important to be cognizant of the educational limitations of the JC to improve clinical and educationally relevant outcomes. Some of these factors are: the artificial setting of the JC which occurs usually in a classroom or conference room, away from the real clinical arena; the risk of heterogeneous participation of JC attendees, promoting passivity in some participants; the lack of motivation to read a complex research paper that does not seem immediately relevant to clinical practice; the difficulty of some of the concepts related to critical appraisal skills, research methods, statistics and evidence-based medicine, which can be hard to grasp and difficult to understand in the JC scenario, where most of the interaction is verbal.

It is noteworthy that the published evidence about JC educational effectiveness in clinically relevant outcomes is limited. Deenadayalan et al. performed an extensive systematic review and found 101 papers, of which 21 fulfilled the inclusion criteria [4], and only 12 described JC effectiveness. Their methodological quality was moderate. More than 80 % of the papers reported that their intervention improved knowledge and critical appraisal skills. Just a few described the psychometric properties of their instrument, and no paper reported evidence of JC impact in clinical practice [4]. There are several initiatives to improve the educational significance and impact of the JC, like the use of e-learning modalities, team-based-learning, problem-based learning, positive-deviant strategies, which could be considered in the implementation of a MEJC [45–55].

We have shown that a continuous professional development activity in the form of a periodic face-to-face Medical Education Journal Club can be maintained in the long-term using evidence-based strategies, and that the experience can have positive effects. The MEJC is an academic activity that can improve the use and appraisal of the medical education literature and promote socialization and community building in health professions’ educational settings.

## Conclusion

A MEJC is an educational activity that can be included in the routine scholarly activities of a medical school department, be a useful adjunct to improve the scholarship of teaching and promote evidence-based medical education practice.
